# SBM–Attention U-Net: A Hybrid Transformer Network for Liver Tumor Segmentation in Medical Images

**DOI:** 10.3390/s26061851

**Published:** 2026-03-15

**Authors:** Yiru Chen, Xuefeng Li, Yang Du, Hui Jiang, Xiaohui Liu, Nan Ma, Xuemei Wang

**Affiliations:** 1 State Key Laboratory of Digital Medical Engineering, School of Biological Science and Medical Engineering, Southeast University, Nanjing 210096, China; 2 Joint Graduate School of Southeast University-Monash University, Suzhou 215123, China; 3 Key Laboratory of Molecular Imaging, Institute of Automation, Chinese Academy of Sciences, Beijing 100190, China

**Keywords:** liver cancer, medical images segmentation, attention mechanism, deep learning

## Abstract

This study proposes a novel liver and liver tumor segmentation model. The architecture integrates BiFormer into the bottom two layers of the Attention U-Net encoder to enhance global semantic context modeling and establish long-range pixel-wise dependencies. The proposed spatial-channel dual attention (SCDA) mechanism is incorporated into the first three encoder layers to refine the fine-grained feature processing capabilities, particularly for precise delineation of liver and tumor boundaries. Eventually, a Mix Structure Block (MSB) is implemented within the decoder to optimize fusion of deep semantic and shallow spatial features, thereby elevating segmentation accuracy. Ablation experiments were conducted on three publicly available datasets. On the 3Dircadb dataset, the mean dice coefficient achieved was 0.9377 and the mean IoU Index achieved was 0.8889. On the LITS dataset, the mean dice coefficient achieved was 0.9257 and the mean IoU Index achieved was 0.8704. On the CHAOS dataset, the mean dice coefficient achieved was 0.9611 and the mean IoU Index achieved was 0.9259. These results validate the functionality and effectiveness of the proposed network model. This study constructed a novel neural network based on attention mechanisms; by enabling precise and automated segmentation directly from raw sensor-acquired medical images, the proposed method enhances the diagnostic value of these imaging sensors, facilitating more accurate clinical decision-making.

## 1. Introduction

Liver cancer is one of the most aggressive and deadly malignancies worldwide, with a 5-year overall survival rate of less than 20%. It is the 3rd leading cause of cancer-related deaths globally, resulting in over 900,000 deaths annually [1]. Chronic viral hepatitis is the most important causative factor for liver cancer, especially chronic infection with the hepatitis B virus and hepatitis C virus. Long-term persistent viral infection leads to recurrent inflammation and damage to the liver, which in turn may progress to liver fibrosis, cirrhosis, and ultimately significantly increase the risk of developing liver cancer. Long-term progression of a variety of chronic liver diseases can lead to the destruction of the liver’s structure and the development of cirrhosis. Cirrhosis itself is a precancerous state in which liver cells are prone to cancer. Chronic excessive alcohol consumption is also a definite risk factor. Alcohol and its metabolites are directly toxic to liver cells and can lead to alcoholic fatty liver, alcoholic hepatitis, alcoholic liver fibrosis, and eventually alcoholic cirrhosis, which in turn increases the risk of liver cancer. Other factors include long-term exposure to certain chemicals, certain inherited metabolic diseases, smoking, and diabetes [2].

Despite advances in multimodal treatment approaches, including surgery, chemotherapy, and radiotherapy, the prognosis for liver cancer patients remains poor, with high recurrence and metastasis rates. The precise diagnosis and personalized treatment of tumors at an early stage are crucial to improve the survival rate of patients. Liver puncture biopsy is the gold standard for confirming the nature of liver tumors by obtaining tissue fragments for pathological examination, but the method is invasive to patients and may lead to false-negative results due to an improperly selected sampling location, resulting in serious consequences. With the advancement of medical imaging technology, non-invasive imaging technology is gradually making up for the shortcomings of traditional puncture biopsy, becoming an important pillar of the accurate diagnosis and treatment of liver tumors, and providing an important theoretical basis for doctors to formulate surgical plans. Medical imaging technology mainly includes computed tomography (CT), magnetic resonance imaging (MRI), ultrasound, etc. CT [3] is essentially a tomographic imaging technology; through mathematical reconstruction, based on the principle of X-ray attenuation, when X-rays penetrate the human body from different directions, the computer will reconstruct a cross-sectional image of the human body through the inverse projection algorithm according to the attenuation value of the ray intensity. The final value of each pixel point reflects the difference in density of the tissues at that location. MRI [4] generates tomographic images through the non-invasive phenomenon of magnetic resonance. Its core physical process is based on the behavior of hydrogen protons in a strong magnetic field: the main magnetic field causes the hydrogen protons to be ordered in the direction of the magnetic field. Subsequently, a radiofrequency pulse of a specific frequency excites the protons to deviate from their original orientation. When the radiofrequency pulse is turned off, the protons release energy and generate electromagnetic signals, which are captured by a receiving coil and reconstructed into a grayscale image by mathematical methods such as the Fourier transform. The image contrast is determined by the relaxation properties of the protons: the T1 relaxation time reflects the rate at which the protons return to the original magnetic field direction, and the T2 relaxation time describes the rate at which the protons decay in the transverse magnetization. As critical medical imaging sensors, CT detectors and MRI radiofrequency coils directly determine the quality of acquired data. The performance characteristics of these sensors, such as detector quantum efficiency in CT and coil sensitivity in MRI, will fundamentally influence the image contrast, signal-to-noise ratio, and spatial resolution. These sensor-specific parameters ultimately affect the accuracy of downstream segmentation tasks.

**Figure 1:**
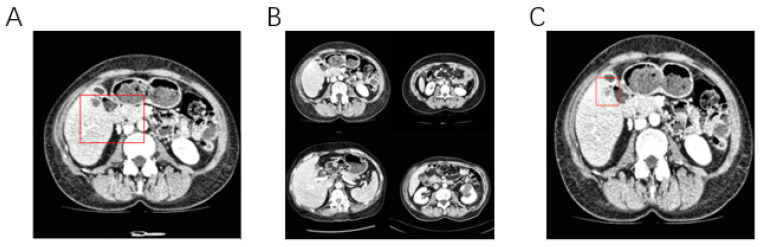
Segmentation of the liver and liver tumors faces many challenges. (**A**) The boundary between the region of interest and the surrounding tissues is ambiguous. (**B**) The individual differences in the liver and liver tumors are very significant. (**C**) Early tumors or small tumors have low contrast with surrounding tissues.

However, even with these sophisticated imaging modalities, segmenting the liver and liver tumors accurately remains challenging due to several factors. As shown in Figure 1, tumors often show infiltrative growth with vague boundaries, adherence to normal liver tissues or blood vessels, and fuzzy boundaries between the liver and adjacent organs. Some early tumors have weak grayscale differences from the surrounding liver parenchyma and low contrast with normal liver tissue [5]. There were significant individual differences in tumor size, shape, location and number [6]. It is difficult to obtain medical image data, and the annotation of liver tumor data needs to be performed by manual outlining by radiologists, which is time-consuming and costly. Doctors also face the problems of subjectivity and poor repeatability in manually outlining the tumor and the region of interest (ROI) [7]. Cross-validation by multiple experts is often required to ensure labeling consistency and reduce labeling subjectivity. At the same time, there are privacy issues with medical image data, making it challenging to obtain labeled data [8].

Deep learning algorithms can handle challenges such as vague boundaries between the liver and surrounding organs, variable tumor morphology, and different sizes well and can automatically learn the multi-level features of liver images to significantly improve the segmentation accuracy. Deep learning models such as U-Net [9] and its variants are capable of automatically learning multi-level features from raw images, including local texture and global semantic information. This ability allows for more accurate identification of fuzzy boundaries and heterogeneous structures of tumors, avoiding the limitations of manually designing features [10]. Deep learning methods can deal well with image noise, low inter-tissue contrast, etc., and have strong generalization ability. Meanwhile, model generalization can be further improved by deep learning data enhancement and transfer learning [11]. The clinical value of deep learning segmentation methods for liver tumor images mainly includes assisted diagnosis, surgical planning, radiation treatment planning and prognosis evaluation [12]. Computer-aided diagnosis technology can provide objective quantitative data for doctors and reduce the rate of missed diagnosis, and at the same time, it can reduce the cost of learning medical images for doctors. With the development of deep learning algorithms and GPU hardware, more and more image segmentation models have been proposed, and the accuracy of some of the algorithms has reached the level of professional physicians, but the existing algorithms still have problems such as an excessive amount of parameters and a loss of semantic information [13]. Therefore, further development of automated and high-precision liver tumor segmentation algorithms is of great clinical significance.

The SBM–Attention U-Net Model proposed in this study combines the advantages of a convolutional neural network (CNN) architecture and multiple attention mechanisms. Attention U-Net [14] is selected as the baseline model of the SBM–Attention U-Net model, which has shown good performance in the field of medical image segmentation. Attention U-Net is an improved U-Net architecture, as vanilla U-Net fuses shallow and deep features through an encoder–decoder structure and skip connections, but may focus excessively on irrelevant regions [15], especially when the target region is small or has low contrast with the background. The U-Net structure is a multi-level cascaded CNN, where the cascade framework extracts ROI and densely predicts specific ROI. All models using the cascade repeatedly extract similar low-level features. This approach leads to redundant use of computational resources and model parameters. Attention U-Net enhances the model’s ability to focus on key regions in an image by introducing an attention mechanism, which is especially suitable for medical image segmentation tasks. Based on the vanilla U-Net, the weights of feature maps are dynamically adjusted to suppress irrelevant background information, thus improving segmentation accuracy and robustness. The main motivation for this study stems from consideration that Attention U-Net currently still has many shortcomings in the task of liver and liver tumor segmentation. Most of the current methods do not provide high accuracy, especially if the tumor is small or irregularly shaped. In this study, we remedied the deficiency of Attention U-Net in predicting liver and liver tumors by introducing various functional attention mechanisms; the advantages and effectiveness of each module will be elaborated in the follow-up content.

The main contributions of this study are as follows:

1. Development of the SBM–Attention U-Net model: The paper develops the SBM–Attention U-Net model for automatic liver and liver tumor segmentation.
2. Convergence of attention mechanisms: This integration utilizes spatial-channel dual attention (SCDA) for improving the model’s fine-grained feature capabilities, BiFormer for global semantic association, and Mix Structure Block (MSB) for fusion of low-order and high-order features.
3. Validity verification: The model was validated using a variety of evaluation metrics on three publicly available datasets, proving the effectiveness and robustness of the SBM–Attention U-Net model.
4. Clinical usefulness: In the early stage of liver tumors, lesions are usually characterized by small size and irregular edges, and the accurate identification of such lesions is of key importance for clinical diagnosis and treatment. This research focuses on improving model sensitivity to small-scale tumors and early-stage lesions, facilitating more effective early clinical screening.

The remaining sections of the paper are organized as follows. Section 2 presents the literature on liver tumor segmentation. Section 3 presents the details of the proposed model. Section 4 describes the training-related configurations. Section 5 presents the evaluation of the proposed model. Finally, Section 6 summarizes the work of this paper and points out the way forward.

## 2. Literature Review

CNNs have now become a major tool for medical image segmentation due to their powerful feature extraction capabilities. Fully Convolutional Networks (FCNs) [16] are a landmark model in deep learning for image segmentation tasks; FCNs achieve pixel-level segmentation through end-to-end training. The core idea of the network is that FCNs replaces the fully connected layer at the end of the traditional CNN with a convolutional layer, which enables the network to accept an input image of any size and to output a segmentation result of the same size as the input size. The FCN gradually restores the spatial resolution lost due to the pooling operation by transposed convolution and restores the low-resolution high-level semantic feature maps back to their original input sizes. FCN also introduces skip connections, which fuse the shallow-layer network with deeper features to fuse the low-level detail information with the high-level semantic information to improve the accuracy of the segmentation boundary. Sun et al. [17] proposed a CT image segmentation method for liver tumors with an improved FCN, which improved the segmentation accuracy by introducing a multi-scale feature-fusion mechanism. Ronneberger et al. proposed the U-Net [9] structure for medical image segmentation in 2015, and U-Net has become the mainstream architecture for liver tumor segmentation due to its special encoder–decoder structure and skip connection mechanism. To further improve the segmentation accuracy, many researchers have proposed various UNet variants. UNet++ [18], proposed by Zhou et al. in 2018, improves on the limitations of vanilla U-Net in medical image segmentation. UNet++ introduces a multi-level nesting structure between the encoder and the decoder, which realizes cross-layer feature fusion through dense connections, making feature fusion richer. Each decoding layer receives features from all corresponding encoder layers, forming a multi-scale feature pyramid. In the output layer, the outputs of multiple layers with the same specifications as the image are weighted and the loss is computed by multiple layers and then updated. Moreover, UNet++ can perform pruning according to the task’s speed requirements. UNet3+ [19] is an improved model of UNet, proposed by Huang et al. in 2020, which further facilitates the fusion of low-level and high-level semantics through full-scale skip connections, fusing lower-order features using different max pooling methods, and fusing higher-order features using different upsampling interpolations to ultimately obtain all the features. In order to extract spatially continuous semantic information in the three-dimensional space of an image, Çiçek et al. proposed 3D U-Net [20], which is specialized for processing 3D images, to extract features in the three dimensions of length, width, and depth, and to capture three-dimensional structural relationships. To solve the problem of gradient vanishing in deep network training while retaining the multi-scale feature-fusion capability of U-Net, Zhang et al. proposed ResUNet [21], which solves the stability problem of U-Net in deep training by introducing residual connections. nnUNet [22] (no-new-Net) is a medical image segmentation framework proposed by Isensee et al. in 2020, which automates the whole process from data preprocessing to hyperparameter selection to reduce human intervention, and at the same time, it generalizes well and adapts to multimodality and multi-resolution medical data. Combining the densely connected design of DenseNet and the symmetric encoder–decoder structure of U-Net, Huang et al. proposed Dense-UNet [23], which utilizes the fact that each layer in Dense Block is connected to all subsequent layers, which enhances the propagation and reuse of features and reduces the number of parameters while alleviating the problem of gradient vanishing. Mahmoodian et al. developed the RESLU-NET [24] method to achieve high-performance liver tumor segmentation under small-dataset conditions through a parameter-efficient transfer learning strategy. Aiming at solving the problem of vague boundaries and complex gradients in liver tumor images, Li et al. proposed Eres-UNet++ [25], which combines an attention mechanism and Res-UNet++, enhances the feature extraction ability of the network, solves the problem of degradation of the deep network, and achieves excellent performance in liver tumor segmentation. Wang et al. combined a squeeze-and-excitation module (SE), Atrous Spatial Pyramid Pooling (ASSP), residual learning, and a U-Net network to propose SAR-U-Net [26], which adaptively extracts image features after each convolution while suppressing irrelevant regions and alleviating the problem of gradient vanishing, which allows the network to increase its depth while still having good accuracy.

All the previously mentioned convolutional neural networks have achieved encouraging outcomes. However, there is a growing trend among researchers to combine attention mechanisms with CNN, following their initial proposal in natural language processing [27] and subsequent introduction to computer vision applications. The attention mechanism enables the model to pay more attention to discriminative regions of the input data by calculating attention scores. The attention mechanism can capture long-range dependencies, breaking through the local receptive field limitations of convolutional operations to establish global contextual associations. The ability of the attention mechanism to dynamically adjust the importance of different regions is useful for dealing with complex structures in medical images. In liver images, tumors may be located in complex backgrounds surrounded by blood vessels and other tissues, and the attention mechanism can help the model focus on the tumor region and reduce misclassification. By visualizing the attention weights, the attention mechanism also has interpretability, allowing one to visualize which regions the model focused on when making predictions. Images are usually two-dimensional matrices of pixels, and the transformer requires a one-dimensional sequence as input. Dosovitskiy et al. first applied the transformer architecture to a computer vision task by proposing vision transformer (ViT) [28], which first segments an image into fixed-size image blocks and then flattens each block into a vector. Each block is flattened into a 16 × 16 × 3 = 768 dimensional vector. These vectors are then linearly projected to obtain a fixed-dimensional embedding vector with positional encoding to preserve spatial information to form the final input sequence, which simply means that the image is segmented into patches and processed into sequential data to capture global contextual information using the self-attention mechanism. But the time complexity of self-attention in ViT is O(N^2^), where N is the number of image blocks. In order to reduce the computational complexity, Liu et al. proposed Swin transformer [29], which performs efficient multi-scale feature learning while reducing the computational complexity through the Shifted Window self-attention mechanism. In order to utilize the unique advantages of U-Net in the field of medical image segmentation, Chen et al. proposed TransUNet [30], which combines the global context modeling capability of a transformer with the local feature extraction capability of U-Net to significantly improve accuracy in medical image segmentation tasks. Sang et al. proposed an across-feature map attention [31] mechanism to specifically address the problem of segmentation of small liver tumors with remarkable results. Diao et al. developed a plug-and-play texture-based automatic pseudo-labeling module [32] to improve liver tumor segmentation performance by using a priori texture information. Despite the encouraging outcomes achieved by the aforementioned CNN-based and transformer-based architectures, several critical challenges remain unaddressed in liver and liver tumor segmentation. CNN-based methods, while effective at extracting local features, struggle to capture long-range semantic dependencies due to their limited receptive fields. Conversely, pure transformer models, despite their superior global context modeling capabilities, suffer from high computational complexity and require large-scale training data. Furthermore, existing attention mechanisms in hybrid models either lack directional sensitivity for handling anatomically oriented structures or fail to adequately fuse multi-scale features, leading to suboptimal performance in segmenting small tumors and those with ambiguous boundaries. To address these limitations, we propose SBM–Attention U-Net, a novel hybrid architecture that synergistically integrates complementary attention mechanisms. The details of the proposed model are elaborated in Section 3.

## 3. Methodology

In this section, each module of SBM–Attention U-Net and its functions will be described in detail.

### 3.1. Baseline Model

**Figure 2:**
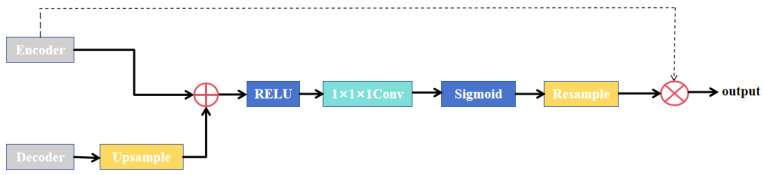
Attention gate structure. Schematic diagram of the attention gate integrated into the skip connections of Attention U-Net. The gate receives two inputs: the gating signal (from the decoder) and the encoder feature map. After upsampling the gating signal to match the spatial dimensions, element-wise summation is performed, followed by ReLU activation and 1 × 1 convolution to generate attention coefficients. A sigmoid function normalizes the coefficients to [0, 1], which are then resampled and multiplied with the encoder feature map to suppress irrelevant background regions and emphasize target structures.

The core architecture of the Attention U-Net is illustrated in Figure 2, where attention gates are integrated into the skip connections of U-Net to autonomously learn importance weights for distinct spatial locations. Features from the encoder are fused with upsampled decoder features through a gating mechanism, generating an attention weight map that accentuates salient regions.

The feature map from the decoder undergoes upsampling to match the spatial dimensions of the corresponding encoder features. These rescaled representations are then combined via element-wise summation, a design choice that amplifies feature responses in target anatomical structures while suppressing noisy activations in irrelevant regions. Although this is computationally more expensive, experiments have shown that it achieves higher accuracy than multiplicative attention.

The subsequent processing involves: (1) Application of ReLU activation to introduce non-linear transformations, enhancing the model’s representational capacity. (2) Dimensionality reduction via 1 × 1 convolution to compress feature channels. (3) By mapping values to the [0, 1] range through Sigmoid activation, background suppression is achieved. This effectively filters the extraneous features and noise in skip connections while reducing both the parameter count and computational overhead of the attention gate. (4) Finally, tri-linear interpolation resamples the features to their original dimensions, yielding attention coefficients. The restored attention coefficients can then be weighted with the feature maps from the encoder, achieving background suppression of the feature maps from the encoder. The attention submodule comprises three fundamental constituents: a query embedding originating from the decoder feature vector, a key embedding deriving from the encoder feature vector, and a value embedding corresponding to the weighted encoder features. Within the Attention U-Net architecture, attention gates are integrated into skip connections. This design harnesses coarse-grained feature maps to acquire higher-level semantic contextual information, thereby suppressing irrelevant features from corresponding encoder layers while simultaneously enhancing model sensitivity and segmentation accuracy for target anatomical regions.

### 3.2. SBM–Attention U-Net

**Figure 3:**
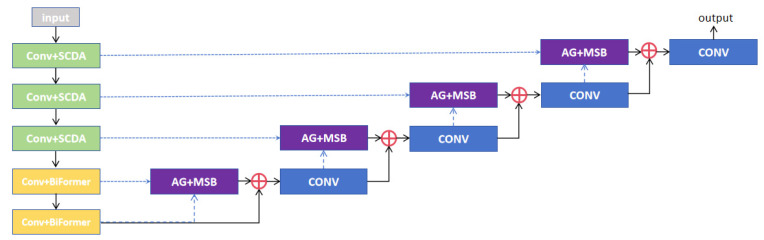
Structure of the SBM–Attention U-Net. The encoder consists of five downsampling stages: the first three stages incorporate SCDA modules for fine-grained low-level feature enhancement, while the two deepest stages employ BiFormer blocks for global semantic modeling. The decoder integrates MSB at each upsampling stage to fuse multi-scale features from skip connections and decoder inputs. Attention gates (AGs) are retained in skip connections to further refine feature transmission.

Through synergistic co-design of hierarchical attention mechanisms and hybrid structural modules, we construct SBM–Attention U-Net, a multi-level feature-fusion framework that progressively integrates local details with global semantics. This architecture seamlessly incorporates advantages from diverse attention paradigms, with its comprehensive structure illustrated in Figure 3.

The SCDA module is deployed in the first three decoder layers to precisely capture fine-grained details within low-order features such as liver edge textures and microcalcifications in tumors, while simultaneously suppressing background-noise interference. Conversely, BiFormer is incorporated into the final two encoder layers to effectively extract global semantic features, including the overall liver morphology and spatial distribution of tumors, thereby addressing the computational redundancy inherent in traditional self-attention mechanisms. The MSB module is strategically implemented in the decoder layers, where it facilitates the fusion of high-order and low-order features through multi-scale convolutional operations and feature-fusion techniques. This design not only preserves local details transmitted via skip connections but also incorporates boundary optimization guided by high-level semantics. Particularly in challenging scenarios such as ambiguous liver boundaries and tumor–vessel interweaving, MSB significantly enhances both the continuity of segmentation boundaries and anatomical plausibility. This architectural strategy capitalizes on the complementary strengths of all modules: SCDA enhances detail localization, BiFormer refines long-range dependency modeling, and MSB achieves multi-scale feature integration.

### 3.3. Low-Order Feature Extraction

Accurate segmentation of liver and tumor structures requires precise capture of low-order spatial features: edges that locate organ boundaries, textures that reveal pathological changes, and point-like structures that indicate tiny tumors. To address this problem, this study proposes an SCDA module for better capturing of low-level features. SCDA is an anatomically inspired architecture that synergistically integrates directional spatial localization and channel-wise feature recalibration. This revolutionary design fundamentally redefines how neural networks perceive and process the critical but vulnerable low-order features that determine segmentation accuracy in liver images. By establishing directional awareness and channel intelligence during initial feature encoding, SCDA fundamentally bridges the cognitive dissociation between artificial neural networks and radiologist-level anatomical comprehension.

In medical image segmentation, the anatomical characteristics of the liver and tumors demonstrate significant directional sensitivity. Nevertheless, the attention mechanism in the vanilla Attention U-Net functions isotropically by assigning uniform weights to features from all directions, which prevents targeted enhancement or suppression of anatomically oriented structures. Consequently, the baseline model demonstrates suboptimal performance when segmenting directionally sensitive structures, particularly in handling irregular tumor boundaries. Furthermore, the lack of spatial and directional awareness may cause attention gates to erroneously amplify noisy regions while suppressing diagnostically relevant detailed features. These limitations frequently lead to the inadequate segmentation of small targets, manifesting as either under-segmentation or false positives. The Coordinate Attention [33] (CA) mechanism effectively addresses these challenges through its spatially conscious design.

**Figure 4:**
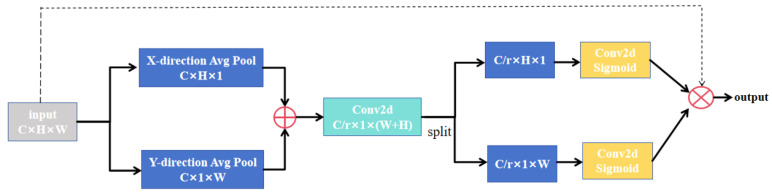
Structure of the CA module. Input feature maps (C × H × W) are pooled along the height and width dimensions separately using global average pooling, producing two directional feature vectors (C × H × 1 and C × 1 × W). These are concatenated and passed through a 1 × 1 convolution for channel reduction, batch normalization, and non-linear activation. The resulting tensor is split back into two directional components, each processed by a 1 × 1 convolution and sigmoid to generate attention weights. These weights are broadcasted and multiplied with the original input to achieve orientation-aware feature recalibration.

The architectural configuration of the CA module is illustrated in Figure 4. The CA mechanism processes input feature maps ([C, H, W]) through dual-path global average pooling along orthogonal spatial dimensions. Initially, width-wise global average pooling generates a feature tensor of size [C, H, 1], effectively compressing spatial information into the height dimension. Simultaneously, height-wise global average pooling produces a [C, 1, W] tensor, compacting information along the width dimension. These operations capture global statistical descriptors along vertical and horizontal orientations respectively. The resulting tensors are concatenated along the spatial axis, forming a unified feature representation of dimensions [C, 1, H + W]. This concatenated feature undergoes channel compression through a 1 × 1 convolutional layer, followed by batch normalization and non-linear activation, yielding the refined representation [C/r, 1, H + W]. The processed feature is then split into two independent branches: [C/r, 1, H] for height-direction weighting and [C/r, 1, W] for width-direction weighting. Each branch undergoes separate 1 × 1 convolution and Sigmoid activation, generating final attention weight maps—spatially specialized matrices of dimensions [1, H, 1] for height-direction weighting and [1, 1, W] for width-direction weighting. These weight maps are subsequently expanded to match the original feature map dimensions through dimensional broadcasting: the height-direction weighting expands along the width axis, while the width-direction weighting expands along the height axis. The final output is produced through element-wise multiplication between the expanded weight maps and the original input features, achieving orientation-aware feature enhancement.

CA achieves explicit spatial position preservation by decoupling channel attention into two parallel one-dimensional feature encoding processes along the horizontal and vertical orientations. The horizontal global pooling operation captures width-wise positional information, thereby enhancing the localization of lateral boundaries. Simultaneously, vertical global pooling extracts height-dimensional spatial relationships, facilitating the identification of superior–inferior boundaries. CA enhances positional information in shallow features, enabling skip connections to transmit more precise spatial information to the decoder. This capability facilitates more accurate reconstruction of the target anatomical structures. In liver and liver tumor segmentation, where both shape and position exhibit substantial inter-case variability, CA improves network robustness by consistently capturing spatial structures across diverse cases. The mechanism’s core advantage lies in leveraging its directional sensitivity to augment the representational capacity of low-level features. By incorporating CA in the early stages, the network preserves critical spatial details that would otherwise be lost during conventional feature extraction. The boundaries of the liver and liver tumors typically exhibit inherently low contrast against surrounding tissues. CA addresses this challenge by enabling more precise localization of hepatic margins, thereby reducing boundary segmentation errors. By enhancing the processing of shallow features, the CA module significantly improves segmentation accuracy, notably for small targets and boundary regions.

Although Attention U-Net employs spatial attention within its skip connections, this mechanism primarily focuses on spatial location significance while neglecting inter-channel relationships. Among these feature channels, certain channels contain critical information for target identification, while others predominantly convey noise or irrelevant information. The upper encoder layers maintain a high spatial resolution with abundant detail preservation but a low semantic abstraction, resulting in substantial redundancy across feature channels. The intensity homogeneity between the liver and adjacent organs may further lead to cross-channel interference. During convolutional operations, the output feature maps are generated by convolving each input channel followed by element-wise summation, assigning equal importance to all channels. This isotropic processing likely drowns out discriminative features while preserving redundant information. Channel attention mechanisms address this limitation by dynamically learning the relative importance of each feature channel, thereby assigning differentiated weights to enhance informative channels while suppressing irrelevant ones. The SE module [34], specializing in channel-wise feature recalibration, presents a particularly suitable solution for mitigating feature interference in multi-target segmentation scenarios.

**Figure 5:**
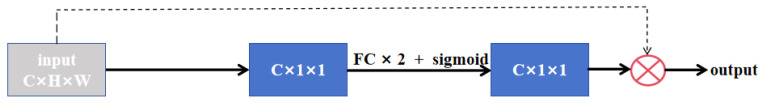
Structure of the SE module. Global average pooling squeezes spatial information from each channel into a channel descriptor (1 × 1 × C). Two fully connected layers (with reduction ratio r) perform excitation: the first reduces dimensionality and applies ReLU, and the second restores the original channel dimensions followed by sigmoid activation. The resulting channel-wise weights are multiplied with the input feature map to adaptively recalibrate feature responses.

As shown in Figure 5, the SE module primarily consists of two sequential operations: squeeze and excitation. The squeeze operation first applies global average pooling to the input feature map, compressing global information from each channel into a scalar value, yielding a 1 × 1 × C vector. This feature vector effectively represents the global contextual information of the input feature map. The excitation operation then processes this vector through two fully connected layers to dynamically learn the importance of each channel. Subsequently, a Sigmoid activation function maps the feature values to the range [0, 1], producing a 1 × 1 × C vector that encodes the relative importance of each channel in the input feature map. This vector is then multiplied element-wise with the original input feature map, generating a recalibrated output where channel-wise features are strategically enhanced or suppressed. The two fully connected layers first reduce and then restore the dimensionality, effectively controlling the model parameters while improving generalization capability. During dimensionality reduction, a hyperparameter r (reduction ratio) is introduced, compressing the 1 × 1 × C vector to 1 × 1 × C/r. The authors of the SE module demonstrated through extensive experimental evidence that r = 16 yields optimal performance. The compressed features undergo non-linear transformation via ReLU activation before being restored to their original dimensionality through a final fully connected layer.

**Figure 6:**
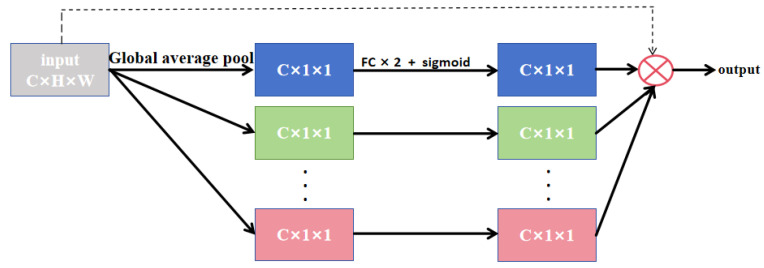
Structure of SEnetV2. After global average pooling, the squeezed vector passes through multiple parallel fully connected branches (dense layers) before aggregation. This design captures richer inter-channel dependencies and improves global context modeling compared to the original SE block.

The upper encoder layers generate feature maps with a high spatial resolution but a low semantic abstraction, making them particularly suitable for SE-based channel enhancement. SENetV2 [35] extends the vanilla SE module by incorporating architectural concepts from ResNeXt, integrating dense connections to strengthen the network’s capacity for modeling inter-channel dependencies and global semantics. This synthesis yields superior feature representations. As illustrated in Figure 6, the squeezed output is processed through multi-branch dense layers before the excitation phase. This design enables more effective learning of diverse input characteristics while explicitly accounting for cross-channel interdependencies during feature transformation.

In contrast to SENet, the SENetV2 architecture facilitates information integration through more extensive layer-wise connections, thereby promoting richer and more diverse learning processes that enable more precise characterization of image details. By recalibrating channel weights, SENetV2 reduces the influence of noise-corresponding channels, thereby mitigating their impact on deeper network layers. Integrating SENetV2 into shallow networks suppresses noise at early feature extraction stages, allowing the network to focus on meaningful anatomical structures and consequently enhancing model robustness. Particularly for small tumors, which may occupy only a few pixels in low-level features, channel reweighting amplifies tumor-specific textures and prevents subtle tumor characteristics from being dominated by channels representing normal liver tissue. This enhancement of the discriminative power of low-level features improves the detection rate of small tumors. The refined low-level features are then delivered to the decoder via skip connections, effectively compensating for the spatial details lost in high-level features and consequently improving the accuracy of boundary segmentation. The SENetV2 module is lightweight, especially in the shallow layers of the encoder, where feature maps have large spatial dimensions but fewer channels, resulting in only a minimal increase in computational overhead.

**Figure 7:**
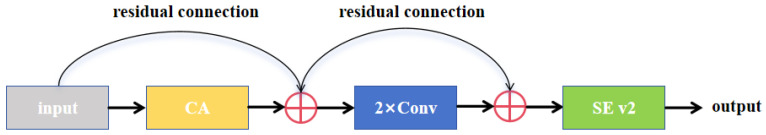
Structure of SCDA. Input features first pass through a CA branch to encode direction-sensitive spatial information. The output is then processed by two consecutive convolutional layers (Conv + BN + ReLU) to refine representations, followed by a SENetV2 block for channel-wise recalibration. Residual connections add the original input to the final output to preserve information and facilitate gradient flow.

As shown in Figure 7, leveraging the complementary advantages of CA and SENetV2, we propose the SCDA module, an innovative feature-enhancement architecture specifically designed to address three core challenges in low-level feature processing for medical image segmentation: loss of spatial positional information, insufficient directional sensitivity, and channel feature redundancy. This module employs a dual-branch collaborative architecture that significantly enhances the accuracy and robustness of liver and tumor segmentation through spatially and channel-wise attentive feature refinement. The input features are first processed by the CA module. The output of the CA module is then passed through two consecutive convolutional layers, each comprising convolution, batch normalization, and activation operations. These transitional layers serve to further transform the CA-output features spatially and channel-wise, enhancing the representational capacity of local features. They also act as a bridge that enables more effective collaboration between the CA and SENetV2 modules by preparing a richer feature representation. The transformed features are subsequently fed into the SENetV2 module. The entire architecture incorporates residual connections that directly combine the original input with the module output. This design promotes gradient flow during training, prevents information loss, and preserves original feature integrity. Through its dual-dimensional attention approach, the SCDA module effectively combines CA’s strength in capturing directionally sensitive spatial information with SENetV2’s capability in modeling inter-channel relationships. This complementary integration substantially enhances the representational capacity of low-level features, enabling superior performance in segmenting directionally sensitive structures while suppressing noise interference, ultimately leading to improved precision in liver and liver tumor segmentation. The module introduces only a minimal increase in parameters, making it well-suited for deployment in shallow layers where feature maps have large spatial dimensions but limited channel depth.

### 3.4. Global Semantic Adaptive Selection

Deep features focus more on high-level semantics like organ shapes. Directly concatenating these features may introduce noise, and traditional skip connections, which rely solely on local receptive fields, struggle to model long-range semantic dependencies between distant pixels. The attention mechanism provides a global receptive field by computing pairwise feature attention scores across all spatial positions. However, this approach imposes substantial computational burdens and high memory consumption, especially for high-resolution inputs. To reduce computational costs, several methods, such as Local Attention [36], Axial Attention [37], and Dilated Attention [38], have been proposed. These methods constrain the regions for attention calculation to reduce complexity, but they face a critical issue: regions outside the constrained scope may contain highly relevant features and ignoring them can prevent the model from effectively learning from such inputs. Therefore, forcing all queries to focus on fixed regions may not be an optimal strategy.

**Figure 8:**
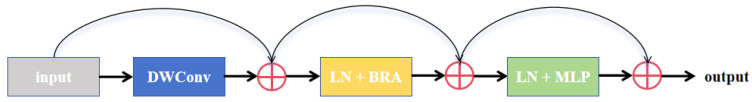
Structure of BiFormer. Input features undergo a 3 × 3 depthwise convolution to encode relative position, then pass through a Bi-level Routing Attention module that selectively aggregates information from the most relevant regions in a content-aware manner. Finally, a two-layer MLP with an expansion ratio e performs per-position embedding. This design efficiently captures global context while reducing computational complexity.

Bi-level Routing Attention [39] (BRA) effectively addresses these challenges through its dual routing mechanism encompassing both region-level and token-level operations. The method first constructs a region-level attention score map, which is then pruned to retain only the top-k connections for each node. This process filters out the majority of irrelevant key–value pairs, allowing each region to focus exclusively on its k most relevant routing regions. After identifying these regions of interest, the algorithm proceeds to compute token-to-token attention. Although the key–value pairs are spatially dispersed, they are efficiently gathered to enable dense matrix multiplication compatible with GPU acceleration. BRA preserves a small set of the most relevant key/value tokens for each query in a content-aware manner, achieving an improved computational-performance trade-off. Crucially, it maintains parallelizability without compromising inference speed. Using BRA as a fundamental building block, the authors proposed a new general-purpose vision transformer named BiFormer; the network structure is shown in Figure 8. Its core characteristics include: focusing only on regions most relevant to the current pixel and significantly reducing computational complexity, while capturing both local details and global context to enhance feature representation capacity. Additionally, it adaptively selects important regions based on input content, thereby improving the robustness of feature selection.

The process initiates with a 3 × 3 depthwise convolution that implicitly encodes relative positional information. This is followed by the application of a BRA module for cross-position relationship modeling, and subsequently a 2-layer MLP module with an expansion ratio of e for per-position embedding.

Integrating BiFormer into the two deepest skip connections of the Attention U-Net yields the following key improvements. By dynamically filtering and fusing features, BiFormer enables more precise feature selection and integration, effectively suppressing noise from the deep encoder layers that is irrelevant to the current decoding task. It adaptively adjusts feature weights to enhance attention toward segmentation targets. The sparse attention mechanism captures global context and establishes long-range semantic dependencies between pixels. The deepest encoder features contain high-level semantic information that directly influences the segmentation accuracy of the decoder. Since the feature maps at the deepest skip connections have relatively a low spatial resolution, BiFormer’s sparse attention remains computationally feasible at this scale. Deploying it only in the two deepest layers strikes an optimal balance between performance and efficiency. Compared to traditional self-attention, BiFormer can process broader contextual information under the same computational budget, thereby improving segmentation performance with manageable computational cost. Last but not least, the content-aware sparse attention mechanism in BiFormer adaptively focuses on semantically relevant regions, making it particularly suitable for processing multimodal sensor data where the underlying feature distributions may differ substantially. This adaptability reduces the need for sensor-specific fine-tuning.

### 3.5. Feature Filtering and Fusion

Traditional CNNs have dual limitations in medical image segmentation: their inherent local receptive field constraints lead to inadequate capture of pixel-level contextual information and some of the improved schemes for expanding the receptive field fail to efficiently model the multi-scale characteristics of images. Although the transformer architecture has the capability of global context modeling, it faces high computational cost and training complexity due to the increase in the number of parameters. To address these challenges, Lu et al. [40] proposed the MSB, which combines a multi-scale parallel large convolution kernel module and an enhanced parallel attention module in a sequential manner. Although initially designed for low-level image dehazing tasks, both the multi-scale large-kernel convolution module and the parallel attention module effectively enhance feature-fusion capabilities. As a result, this block is particularly suitable for integration into the decoder of Attention U-Net, where it facilitates the improved fusion of features from both the encoder and the decoder.

**Figure 9:**
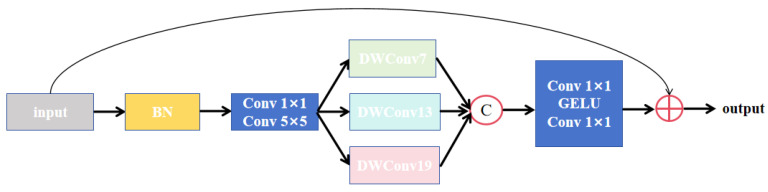
Structure of the multi-scale parallel large convolution kernel module. Input features are normalized and split into two parallel paths: a 1 × 1 convolution for channel adjustment and a 5 × 5 convolution to allow a larger receptive field. The outputs are fed into three parallel depthwise dilated convolutions with different dilation rates to capture multi-scale context. These features are concatenated, refined by 1 × 1 convolutions and GELU activation, and finally added to the input via a residual connection.

An illustration of the multi-scale parallel large convolution kernel module’s structure is provided in Figure 9. The input feature map first undergoes batch normalization to accelerate training convergence and enhance model stability. It then passes through two parallel convolutional layers: a 1 × 1 convolution for channel dimensionality adjustment and a 5 × 5 convolution to capture a relatively larger receptive field. The convolved feature maps are subsequently fed into three parallel depthwise dilated convolutional layers with different dilation rates, enabling the extraction of multi-scale features. The outputs of these dilated convolutional layers are concatenated and processed sequentially by a 1 × 1 convolution, a GELU activation function, and another 1 × 1 convolution. The two 1 × 1 convolutions further refine channel dimensions and integrate features, while the GELU activation introduces non-linear expressive capacity. Finally, a residual connection adds the processed output to the original input feature map, promoting gradient propagation and mitigating vanishing gradient issues.

As the largest organ in the abdominal cavity, liver segmentation requires the simultaneous consideration of global morphological localization and detailed local delineation. Tumor segmentation, on the other hand, faces challenges such as substantial size variation and high heterogeneity. The MSB addresses these issues by deploying convolutional kernels with different receptive fields in parallel, enabling the simultaneous extraction of low-resolution global semantic features and high-resolution local detailed features. This multi-scale feature-fusion mechanism allows the decoder to preserve the high-level semantic information transferred from the encoder while restoring spatial resolution. Additionally, it enhances sensitivity to small tumors or complex boundaries through local feature refinement, thereby achieving dual optimization in liver contour delineation and tumor lesion localization.

**Figure 10:**
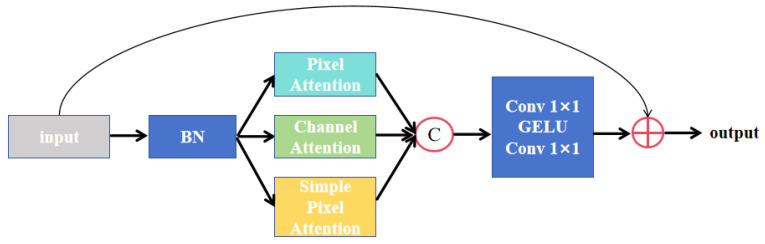
Structure of enhanced parallel attention module. Input features are normalized and processed by three parallel attention branches: pixel attention (focuses on spatial positions), channel attention (models channel dependencies), and simple pixel attention (models local pixel correlations). The outputs of these branches are aggregated to refine feature representation, improving the segmentation of boundaries and small structures.

The structure of the enhanced parallel attention module is illustrated in Figure 10. The input feature map first undergoes batch normalization to improve the stability of the model. It is then processed collaboratively through three parallel attention branches, with each branch dedicated to extracting and refining information from different dimensions. The pixel attention module primarily captures local features related to spatial positions, anchoring key anatomical landmarks. The channel attention module models feature dependencies along the channel dimension, enhancing the recognition rate of tumor-heterogeneous regions. The simple pixel attention module focuses on modeling local correlations between adjacent pixels, which helps improve the continuity of tumor boundaries.

This enhanced parallel attention mechanism complements the attention gate in Attention U-Net. While the attention gate primarily suppresses redundant information in skip connections, the attention mechanism within the MSB further refines feature selection inside the decoder. It significantly improves segmentation accuracy and robustness, particularly for tumors with blurred boundaries and lesions intertwined with blood vessels. Integrating the MSB into the decoder layers of Attention U-Net constructs a feature-fusion framework that progresses from global to local and from coarse to fine granularity.

## 4. Experiments

To validate the effectiveness of the proposed deep learning framework, this study will conduct a series of ablation experiments. Through these experimental validations, the research will be able to evaluate the accuracy, robustness, and applicability of the proposed framework, thereby providing theoretical foundations and technical support for its promotion and development in clinical applications.

In this section, we first introduce the datasets used for the experiments. Then we will clarify the data preprocessing processes. Finally, we will describe experimental settings such as the evaluation metrics, the loss function, etc.

### 4.1. Input Datasets

This study was evaluated on three publicly available liver datasets. An identical 6:2:2 random division for the training, validation, and test sets was applied uniformly to every ablation run to eliminate data distribution bias.

#### 4.1.1. 3D Image Reconstruction for Comparison of Algorithm Database (3Dircadb)

The 3Dircadb [41] dataset, released by the French IRCAD Institute, is an abdominal medical imaging collection primarily designed for the development and validation of liver and intrahepatic tumor segmentation algorithms. This database comprises 3D CT scans from 10 female and 10 male patients, with a liver tumor incidence rate of 75%. The data is organized into 20 directories corresponding to 20 distinct patients. All images are provided in DICOM format and are accessible for analysis using standard medical imaging processing software.

#### 4.1.2. Liver Tumor Segmentation Challenge (LITS)

The LITS2017 [5] dataset is a medical imaging dataset designed for liver and tumor segmentation. It contains a substantial number of CT scans and aims to promote the development of automated segmentation methods for the liver and liver tumors. This dataset was jointly released by institutions including Fuwai Hospital, the Chinese Academy of Medical Sciences, and Columbia University Medical Center. LITS2017 includes medical imaging data from 130 cases selected in 2017, comprising CT scans along with corresponding segmentation labels of the liver and tumors. These annotations facilitate the development of novel liver and tumor segmentation algorithms and enable the performance evaluation of such methods. The image data are stored in the nii format and can be accessed and analyzed using medical imaging processing software.

#### 4.1.3. Combined (CT-MR) Healthy Abdominal Organ Segmentation (CHAOS)

The CHAOS [42] dataset represents a classic benchmark in abdominal medical image segmentation. Its most notable feature is the provision of paired multimodal CT and MRI data along with corresponding annotations. Originally released during the ISBI 2019 Challenge, the dataset comprises 40 paired CT and MRI cases, among which only 20 include publicly available annotations for training purposes. All imaging data are provided in DICOM format. The CHAOS dataset includes abdominal MRI scans with ground-truth annotations for four organs: the spleen, liver, left kidney, and right kidney. For the purpose of this study, only the annotated MRI data containing labels for these four organs were utilized.

### 4.2. Data Preprocessing

Windowing technique: The windowing technique effectively calibrates the raw sensor outputs to enhance clinically relevant features. This process compensates for variations in sensor sensitivity across different CT scanners, ensuring consistent image presentation regardless of the acquisition device. CT images are stored in computers using Hounsfield unit (HU) values, which are device-independent, meaning that CT images from different hospitals can undergo identical preprocessing. The HU is a standardized measure for quantifying tissue density in CT images, typically referenced to water. The HU value of a tissue depends on its density and generally spans a wide range, resulting in inherently low image contrast. Windowing primarily adjusts the grayscale values of CT images through two key parameters: Window Center (WC) and Window Width (WW). The WC represents the midpoint of the displayed pixel values. Decreasing the WC brightens the image while increasing it darkens the image. The WW defines the range of pixel values displayed within the window. By adjusting the WC and WW, specific HU ranges corresponding to different tissue types can be selectively enhanced, thereby optimizing image interpretability and diagnostic efficacy. The primary objective of windowing is to improve image contrast to more clearly visualize tissues of varying densities. For instance, setting the WC within the soft tissue HU range allows clinicians to more easily identify pathologies such as tumors or inflammation, while adjusting the window to focus on the bone HU range aids in detecting fractures or other skeletal abnormalities. Appropriate window settings enable more accurate analysis of CT images, leading to improved diagnostic accuracy. Additionally, window processing can reduce the impact of artifacts by optimizing the visualization of HU values. Proper window configuration enhances overall image clarity, making artifacts less prominent in the displayed result. The flexibility of window adjustments allows radiologists to swiftly switch between different tissue types, ensuring optimal display for each anatomical structure and significantly improving analytical efficiency.

Histogram equalization: Noise may obscure critical clinical information, leading to misdiagnosis or missed diagnoses. CLAHE (Contrast-Limited Adaptive Histogram Equalization) is an image processing technique designed to enhance image contrast while preventing excessive amplification of noise. CLAHE adaptively enhances local contrast, mitigating effects such as non-uniform sensor illumination. In OpenCV, a CLAHE object can be created using the cv2.createCLAHE() function, and the image can be equalized by calling the apply() method of this object. This function has two parameters: clipLimit and tileGridSize. The former controls contrast limiting—when the histogram of a local region exceeds a preset threshold, CLAHE clips it and redistributes the pixel values to prevent noise amplification. The latter divides the image into tiles and performs histogram equalization within each block. The equalized sub-regions are then reassembled to form the final enhanced image. This local processing approach enhances details and edges in the image, making subtle lesions and structures more visible.

Black-border cropping: This process removes excessive content-free areas around the edges of the image to reduce computational load and accelerate convergence.

Slicing: When the dataset consists of 3D data, it is sliced into 2D images to adapt to model input requirements.

Normalization: This step accelerates model convergence, helps prevent gradient vanishing and explosion issues, and avoids distribution shifts in features caused by minor data perturbations, thereby improving prediction stability.

Sharpening: This enhances high-frequency information in the image, thereby increasing the model’s sensitivity to critical features. By emphasizing object contours and fine structures, it helps neural networks more clearly capture task-relevant patterns.

### 4.3. Training Configuration

To isolate the performance contribution of each specific architectural modification, the modules were integrated sequentially. Across all model variants, the hyperparameter configurations were kept strictly constant. It is important to note that all models evaluated in this study, including the baseline Attention U-Net and all SBM–Attention U-Net variants, were trained entirely from scratch. No pre-trained weights were utilized for network initialization, ensuring that the observed performance improvements are strictly attributable to the proposed architectural innovations rather than external prior knowledge. The model configuration utilizes Synchronized Batch Normalization instead of traditional batch normalization to maintain statistical consistency in multi-GPU distributed training scenarios, significantly improving training stability. The decoder head incorporates a composite loss function designed to optimize both pixel-wise classification accuracy and region overlap. Specifically, we combine a weighted Cross-Entropy Loss and a weighted Dice Loss. These two components are defined separately and then summed to form the total training objective.

The Cross-Entropy Loss (L_CE_) ensures precise pixel-level classification and enhances boundary discrimination. It is defined as:

(1) $$L_{CE}=-\frac{1}{N}\sum_{i=1}^{N}\left[y_{i}\log\left(p_{i}\right)+\left(1-y_{i}\right)\log\left(1-p_{i}\right)\right]$$

where N is the total number of pixels, y_i_ is the ground-truth label (0 or 1), and p_i_ is the predicted probability for pixel i belonging to the foreground.

The Dice Loss (L_Dice_) mitigates class imbalance by maximizing the overlap between the predicted and ground-truth regions. It is defined as:

(2) $$L_{\mathrm{Dice}}=1-\frac{2\sum_{i=1}^{N}\left(y_{i}p_{i}\right)}{\sum_{i=1}^{N}y_{i}+\sum_{i=1}^{N}p_{i}}$$

The total loss (L_total_) is a weighted sum of these two components:

(3) $$L_{\mathrm{total}}=\lambda_{1}\cdot L_{\mathrm{CE}}+\lambda_{2}\cdot L_{\mathrm{Dice}}$$

where $\lambda_{1}$ and $\lambda_{2}$ are weighting coefficients. In our implementation, we set $\lambda_{1}=2.0$ and $\lambda_{2}=2.0$ to balance the contribution of each term. Thus, the final loss function used for optimization is:

(4) $$L_{\mathrm{total}}=-\frac{2}{N}\sum_{i=1}^{N}\left[y_{i}\log\left(p_{i}\right)+\left(1-y_{i}\right)\log\left(1-p_{i}\right)\right]+2-\frac{4\sum_{i=1}^{N}\left(y_{i}p_{i}\right)}{\sum_{i=1}^{N}\left(y_{i}+p_{i}\right)}$$

This combined loss design facilitates stable gradient updates during training and addresses both the need for precise boundary delineation and robust performance under class imbalance.

The experiments were implemented using PyTorch 1.10.0+cu113. To ensure consistent hyperparameter control across ablation studies, MMSegmentation v0.29.1+ and OpenCV v4.10.0 were adopted. The model was trained on four RTX 3090 GPUs. Input images were uniformly randomly cropped to a 256 × 256 resolution, and flipping was disabled to preserve the anatomical orientation consistency of the liver. Zero-padding was applied to maintain spatial structural integrity. During training, photometric distortion was introduced to simulate varying imaging conditions, enhancing model robustness to brightness and contrast variations. No data augmentation was applied during testing. The Adam optimizer was used with an initial learning rate of 1 × 10^−4^ and momentum decay coefficients β_1_ and β_2_ set to 0.9 and 0.999, respectively. A polynomial learning rate decay strategy (power = 0.9) was employed, which achieved a smoother convergence curve than traditional gradient descent methods over 20,000 maximum iterations. The minimum learning rate was set to 1 × 10^−5^ to avoid oscillation. To prevent overfitting, layer normalization was used in place of batch normalization in parts of the backbone network. Notably, the find_unused_parameters = True option was enabled to accommodate dynamic computation graphs and ensure complete gradient propagation through attention modules.

The model evaluation metrics mainly include IoU, precision, recall, and dice.

IoU measures the degree of overlap between the predicted region *A* and the actual region *B*, with a value range of [0, 1]. The higher the value, the closer the prediction is to the actual result. The IoU calculation formula is as follows:

(5) $$\mathrm{IoU}\left(A,B\right)=\;\frac{\left|A\cap B\right|}{\left|A\cup B\right|}$$

The absolute value symbol refers to the number of pixels.

Precision refers to the proportion of actual positive classes in the predicted positive class region, which measures the accuracy of the prediction. Precision answers the question “How many of the regions predicted to be liver/tumor are correct?”. The precision calculation formula is as follows:

(6) $$\mathrm{Precision}\left(A,B\right)=\;\frac{\left|A\cap B\right|}{\left|A\right|}$$

Recall measures the proportion of actual positive cases that are correctly predicted by the model, reflecting its ability to identify all relevant instances. It answers the question “Of all the true liver/tumor regions, how many were successfully detected?”. The formula for recall is defined as:

(7) $$\mathrm{Recall}\left(A,B\right)=\;\frac{\left|A\cap B\right|}{\left|B\right|}$$

The dice coefficient measures the similarity between two regions, with values ranging from [0, 1], and is more sensitive to small region overlaps. The dice formula is as follows:

(8) $$\mathrm{Dice}\left(A,B\right)=\;\frac{2\cdot \left|A\cap B\right|}{\left|A\right|+\left|B\right|}$$

These evaluation metrics can be used to comprehensively assess the performance of the model in image segmentation tasks.

## 5. Results

Extensive ablation experiments were conducted on three public datasets to validate the effectiveness and generalizability of each proposed module. Detailed per-module results are provided in the Supplementary Materials (Tables S1–S15). This section synthesizes the key findings and focuses on the clinically significant improvements achieved by the complete SBM–Attention U-Net.

### 5.1. Result in 3Dircadb

**Figure 11:**
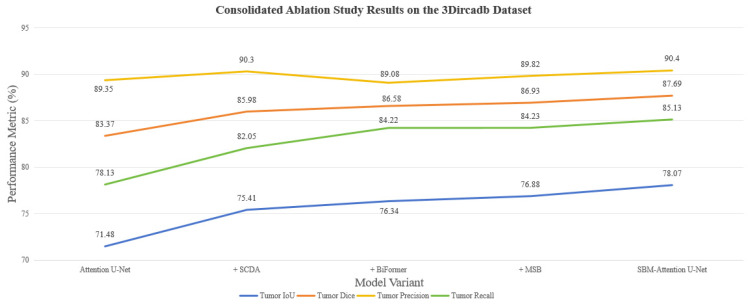
Trends of the consolidated ablation study results on the 3Dircadb dataset. The line chart illustrates the performance changes in tumor segmentation after progressively integrating different modules into the baseline Attention U-Net. The sequential integration of the proposed modules yielded consistent and significant performance gains.

To clearly demonstrate the incremental contributions of each proposed module, Table 1 and Figure 11 consolidates the key performance metrics of the baseline model and its variants on the 3Dircadb dataset. The sequential integration of the proposed modules yielded consistent and significant performance gains.

The proposed method achieves a significant breakthrough in liver tumor segmentation on the 3Dircadb dataset. Experimental results demonstrate systematic improvements across key tumor segmentation metrics: the dice coefficient rises from 83.37% to 87.69%, indicating markedly optimized global consistency in tumor segmentation and more precise differentiation between tumors and surrounding healthy tissue. Recall improves from 78.13% to 85.13%, an increase of 7.00%. The improvement in tumor recall holds significant clinical importance by directly increasing the detection rate of tumor-bearing cases and reducing missed diagnoses. This represents an essential advancement for early-stage liver cancer screening. Global performance is also consistently enhanced: the mean dice score increases from 91.58% to 93.77%, reflecting more balanced performance across the entire segmentation task. Importantly, background segmentation maintains high precision, with background IoU rising from 99.61% to 99.7%, confirming that the improvements do not introduce additional noise or compromise the stability of background segmentation. This further validates the robustness of the model.

The performance improvement stems from the synergistic mechanism of the three modules. The SCDA module significantly enhances the ability to capture textural features at tumor boundaries in shallow encoder layers through its dual channel-space attention mechanism. The BiFormer module establishes global semantic correlations in deeper encoder layers, optimizing the topological relationship modeling between tumor regions and liver parenchyma, thereby elevating the tumor IoU to a new high of 78.07%. Meanwhile, the MSB effectively resolves feature misalignment issues in skip connections via multi-scale feature reorganization, enabling the tumor dice coefficient to reach 87.69% while simultaneously raising background segmentation accuracy to a peak value of 99.70%. It is noteworthy that tumor precision increased from 89.35% to 90.40%, indicating that the model effectively controls false-positive growth while improving sensitivity.

**Figure 12:**
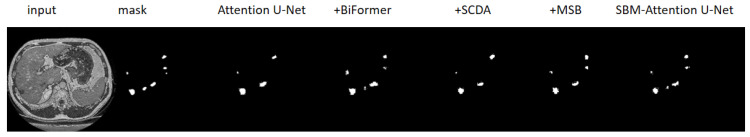
Model inference results on 3Dircadb dataset, where the white areas represent liver tumors and the black area represents the background.

Figure 12 illustrates the segmentation inference results of the model on the 3Dircadb dataset. This synergistic mechanism enables the model to meet the threshold for clinical utility. The result validates that multi-module collaboration systematically enhances tumor structure recognition capabilities, providing a new technical solution for liver cancer imaging diagnosis.

### 5.2. Result in LITS

**Figure 13:**
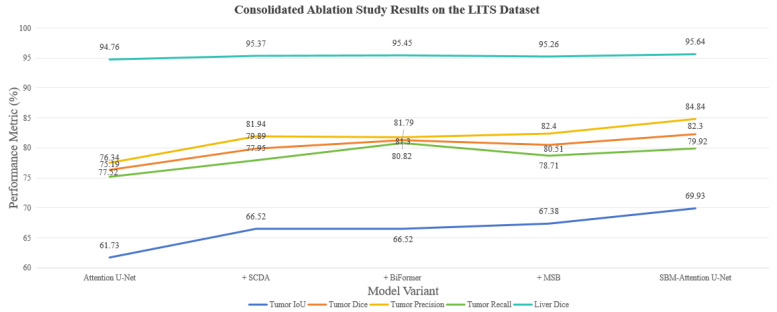
Trends of the consolidated ablation study results on the LITS dataset. The line chart demonstrates the performance evolution for both tumor and liver segmentation across different model configurations.

Table 2 and Figure 13 present the consolidated ablation results on the more challenging LITS dataset, which includes liver parenchyma segmentation.

This multi-module collaborative improvement strategy achieves breakthrough performance on the LITS dataset across multiple dimensions. Specifically, tumor segmentation metrics show significant progress: IoU increases by 8.2 percentage points, indicating substantially improved spatial overlap between segmentation results and ground-truth annotations, which provides critical value for clinical surgical planning such as resection margin definition. The dice coefficient improves by 5.96%, demonstrating more accurate global consistency in tumor boundary delineation. Recall rises from 75.19% to 79.92%, reflecting a marked enhancement in the model’s ability to detect low-contrast lesions. Concurrently, liver parenchyma segmentation performance is also optimized, the liver dice coefficient increases from 94.76% to 95.64%, a further gain of 0.88% on an already high baseline. The precision rate rises from 94.35% to 95.52%, indicating that the model not only reduces missed detections but also further decreases the risk of misclassifying other tissues as liver. The dual improvement in the liver and tumor dice coefficients enables more accurate volume calculation and boundary guidance for surgical procedures such as hepatic segment resection and radiofrequency ablation. In terms of global evaluation metrics, the background dice score increases from 99.72% to 99.76%, achieving a 0.04% gain even at near-perfect accuracy levels. These results confirm that the model effectively distinguishes target structures from background noise without introducing additional interference through feature fusion, further validating the enhancement of the model’s overall robustness. The LITS dataset aggregates data from multiple clinical centers, featuring diverse scanner vendors and protocols. The model’s state-of-the-art performance on this dataset is strong evidence of robustness.

**Figure 14:**
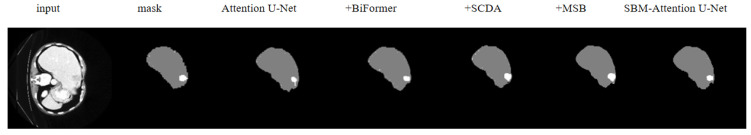
Model inference results on LITS dataset, where the white areas represent liver tumors, the gray area represents the liver, and the black area represents the background.

Figure 14 illustrates the segmentation inference results of the model on the LITS dataset. The experiments validate that these improvements systemically enhance the overall model performance, with notably balanced cross-category performance and especially higher segmentation consistency in multi-target scenarios. This hierarchical optimization strategy provides an innovative solution for medical image segmentation, delivering remarkable advancements in addressing the challenging task of liver tumor segmentation.

### 5.3. Result in CHAOS

**Figure 15:**
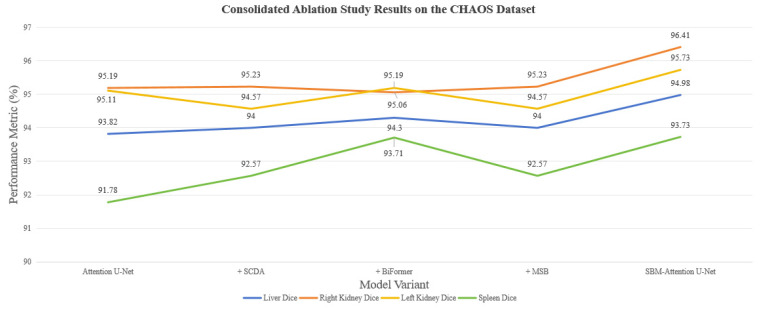
Trends of the consolidated ablation study results on the CHAOS dataset. The line chart illustrates the performance evolution of multi-abdominal organ segmentation (liver, right kidney, left kidney, and spleen) across different model configurations. The sequential integration of the proposed modules demonstrates comprehensive optimization across all target categories.

The effectiveness of our model for multi-abdominal organ segmentation is evaluated on the CHAOS dataset. Table 3 and Figure 15 provide a complete overview of the performance for all annotated organs across different model configurations.

This collaborative strategy achieves milestone performance breakthroughs in the CHAOS multi-organ segmentation task, significantly enhancing the segmentation accuracy of multiple abdominal organs, with particularly outstanding results for anatomically complex and morphologically variable organs such as the spleen and liver. Key metric comparisons demonstrate groundbreaking progress in liver segmentation: the liver dice coefficient increases by 1.16% and the IoU improves by 2.09%, effectively enhancing the model’s ability to comprehend the overall liver morphology. The spleen segmentation dice coefficient jumps from 91.78% to 93.73%. The right kidney segmentation achieves a dice score of 96.41%. The overall performance shows a leap forward: the mean dice coefficient improves by 1% and the mean IoU increases by 1.80%, reflecting comprehensive optimization across all five target categories and especially higher segmentation consistency in multi-organ coexistence scenarios. The high performance on the kidneys and spleen suggests the model generalizes well to organs with intensity distributions similar to the liver.

**Figure 16:**
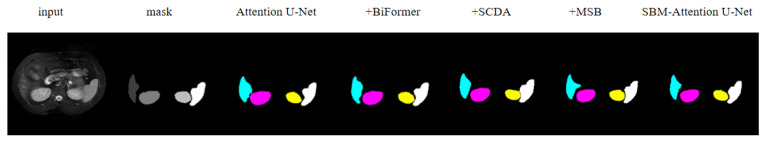
Model inference results on CHAOS dataset, the blue area represents the liver, the purple area represents the right kidney, the yellow area represents the left kidney, and the white area represents the spleen.

Figure 16 illustrates the segmentation inference results of the model on the CHAOS dataset. Experimental results confirm that the three modules respectively address core challenges in medical image segmentation: feature extraction, semantic modeling, and fusion mechanisms. Collectively, they advance multi-organ segmentation accuracy. The proposed improvement scheme achieves dual enhancement in precision and robustness for medical image segmentation tasks, establishing a novel paradigm for multi-target segmentation in abdominal medical imaging.

To evaluate the statistical significance of the performance improvements between the baseline Attention U-Net and the proposed SBM–Attention U-Net across the 3Dircadb, LITS, and CHAOS datasets, a Wilcoxon signed-rank test was conducted on the sample-level evaluation metrics using the Python scipy library (v1.17.0). This non-parametric test was selected to account for the high inter-patient variance typical in medical imaging datasets. The analysis yielded *p* < 0.05 for all three datasets, indicating statistical significance. This confirms that the observed performance gains are consistent across the test cohorts rather than being artifacts of data variance or extreme outliers.

### 5.4. Horizontal Comparative Analysis

This chapter presents a horizontal evaluation of the performance of each model based on the latest literature data.

The evaluation on the 3Dircadb dataset highlights the model’s efficacy in handling limited training data, a common challenge in medical imaging. As presented in Table 4, SBM–Attention U-Net achieved a tumor dice coefficient of 87.69%, significantly outperforming the pure transformer-based Swin-UNet. This substantial margin suggests that while pure transformers struggle with inductive bias in low-data regimes, our hybrid approach successfully mitigates this via the SCDA module. Furthermore, the proposed model surpasses the widely used TransUNet and recent 2025 architectures such as E^2^Net and G-UNETR++. These results indicate that the integration of BiFormer enables superior global semantic modeling compared to gradient-enhanced encoders, even when sample sizes are restricted.

Table 5 details the comparative results on the LiTS dataset. SBM–Attention U-Net demonstrates remarkable robustness. Notably, our model outperforms the multi-scale attention network MS-FANet by a significant margin of 8.1% in tumor segmentation. While the State-Space Model SegMamba (V2) shows competitive performance, SBM–Attention U-Net delivers comparable results without the architectural complexity of SSMs.

The generalization capability of SBM–Attention U-Net is further substantiated by the results on the CHAOS dataset (Table 6). This is particularly evident in the segmentation of the spleen, an organ characterized by high anatomical variability and blurred boundaries. Our model achieved a dice score of 93.73%, significantly outperforming both the 2024 nnU-Net variant MRSegmentator and SegMamba (V2). This improvement directly validates the superiority of the SCDA module in handling irregular organ boundaries. Furthermore, SBM–Attention U-Net surpasses the Swin transformer-based model, Swin-UNETR, in both the liver and kidney segmentation metrics.

In conclusion, SBM–Attention U-Net not only overcomes the limitations of single architectures but also establishes new state-of-the-art standards across single-tumor, composite, and multi-organ segmentation tasks, demonstrating its broad applicability in clinical medical image analysis.

### 5.5. Time Complexity

**Figure 17:**
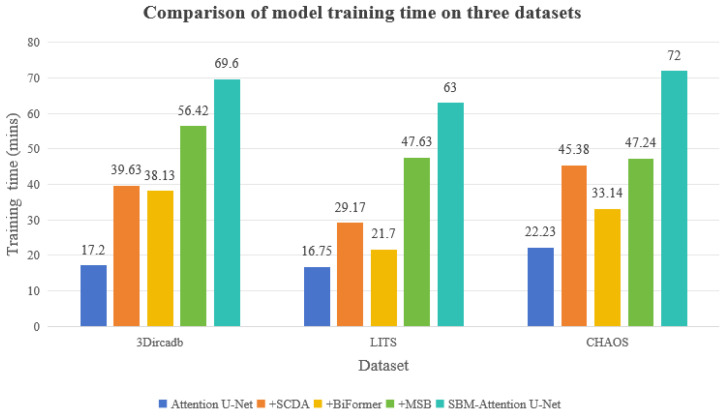
This figure compares the training-time costs of the baseline Attention U-Net, the individual ablation modules (+SCDA, +BiFormer, +MSB), and the proposed SBM–Attention U-Net.

The training-time comparisons across the three datasets are shown in Table 7 and Figure 17.

**Figure 18:**
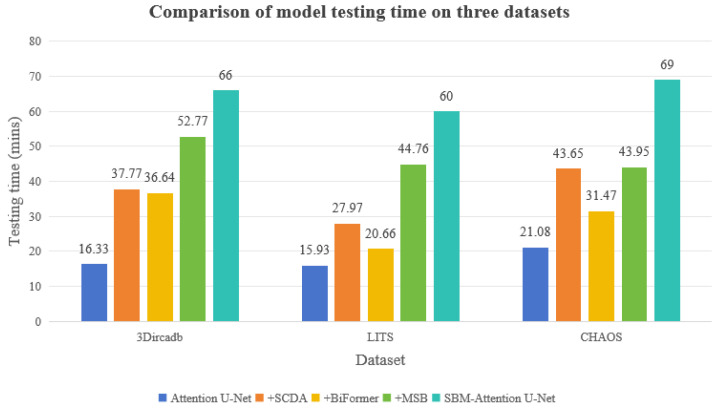
This figure compares the testing-time costs of the baseline Attention U-Net, the individual ablation modules (+SCDA, +BiFormer, +MSB), and the proposed SBM–Attention U-Net.

The testing-time comparisons are presented in Table 8 and Figure 18.

This study systematically evaluates the computational efficiency of the module enhancement strategy. The computational overhead of the model is positively correlated with the structural complexity of the modules, and the increasing trend of time consumption during the testing phase highly aligns with that of the training phase. As shown in the tables, the baseline Attention U-Net demonstrates efficient computational performance across all three datasets. The dual attention mechanism introduced by SCDA significantly increases the computational load, primarily due to the dynamic weight generation process along the channel and spatial dimensions, leading to considerable additional time overhead during training. The global dependency modeling operation of BiFormer exhibits a moderate increase in computational cost on complex datasets, but demonstrates favorable computational optimization characteristics in relatively complex data distributions. The feature-fusion operation of MSB incurs the most substantial time cost, with its cross-scale feature reorganization operation becoming the primary computational bottleneck of the system. When the three modules work collaboratively, the training time remains within an acceptable range for the datasets.

The ultimate value of the SBM–Attention U-Net is determined by its fit within specific clinical workflows. We categorize its utility based on the temporal and accuracy requirements of different procedures. Several days before surgery, surgeons require precise 3D models of the liver, tumor, and vasculature to plan resection planes and calculate the future liver remnant volume. The model is highly useful. The high dice scores ensure accurate volumetric calculations, which is the safety-critical metric for preventing post-hepatectomy liver failure. The high computational time is acceptable in this asynchronous workflow. The model is currently infeasible for intra-operative navigation. With inference times in the range of minutes per slice, the SBM–Attention U-Net cannot support real-time navigation. The lag would result in a dangerous misalignment during surgical manipulation [55]. Surgical navigation requires selective module pruning. Developing a lightweight version for mobile healthcare scenarios through this method can optimize computational efficiency while preserving critical performance gains. When applied to screening and detection, the model is moderately useful. High recall reduces the risk of missing early-stage cancers, but the computational cost makes it expensive to run on every screening scan in a high-throughput hospital setting. Furthermore, the model’s false-positive rate could lead to unnecessary anxiety or follow-up biopsies. A lighter, faster model might be preferred for initial triage, with SBM-Net used as a “second reader” for difficult cases.

## 6. Conclusions and Future Work

This study developed SBM–Attention U-Net, a hybrid transformer–CNN architecture integrating SCDA, BiFormer, and MSB to address critical limitations in liver tumor segmentation. Our model achieved state-of-the-art performance across three public datasets, with tumor dice coefficients reaching 87.69% (3Dircadb), 82.30% (LITS), and 94.98% (liver in CHAOS). The model exhibits robust cross-modal generalization. These results demonstrate significant advancements in capturing fine-grained boundaries, global context, and multi-scale feature fusion.

While this study demonstrates promising outcomes, several methodological refinements warrant further investigation. Model accuracy improvement relies on network architecture complexity with significant expansion of layer depth and parameter size. This design strategy inevitably introduces computational performance bottlenecks. The duration of the training/inferring process increases dramatically, and the memory usage grows significantly. The surge in computational resource demand and the decrease in model interpretability form a double constraint, which seriously hinders the application in clinical scenarios. Subsequent efforts will aim to optimize the model for lightweight deployment and explore direct integration with raw sensor data streams, enabling real-time interactive systems for real-time intra-operative guidance. Although SBM–Attention U-Net achieves excellent performance on three public datasets, segmentation failures may still occur in certain complex clinical scenarios. Although the proposed architecture greatly enhances the overall detection of small tumors, the segmentation of extremely small lesions may still fail. As shown in Figure 11, extremely small lesions occupy only a few pixels in shallow feature maps and are easily diminished by downsampling operations. Although the SCDA module enhances edge-texture representation, it remains challenging to reliably distinguish such tiny targets. Future research can introduce multi-scale feature-enhancement modules to enhance sensitivity to extremely small lesions. When the data contain severe artifacts, future integration with emerging sensor technologies, such as photon-counting CT detectors [56] or high-field MRI systems [57], could further enhance segmentation accuracy. The restricted scale of publicly accessible datasets constrains both data diversity and volume, thereby compromising model robustness. Subsequent research should explore semi-supervised learning paradigms to develop liver tumor segmentation networks trained on limited annotated data. Additionally, generative adversarial networks and diffusion models offer viable pathways for synthetic data augmentation to enhance sample heterogeneity and deal with atypical anatomy.

## Figures and Tables

**Table 1:** Consolidated ablation study results on the 3Dircadb dataset. Bold represents optimal performance in its evaluation metric.

Model Variant	Tumor IoU	Tumor Dice	Tumor Precision	Tumor Recall	Mean Dice	Mean Recall
Attention U-Net	71.48	83.37	89.35	78.13	91.58	89.01
+SCDA	75.41	85.98	90.30	82.05	92.91	90.97
+BiFormer	76.34	86.58	89.08	84.22	93.21	92.04
+MSB	76.88	86.93	89.82	84.23	93.39	92.05
SBM–Attention U-Net	**78.07**	**87.69**	**90.40**	**85.13**	**93.77**	**92.51**

**Table 2:** Consolidated ablation study results on the LITS dataset. Bold represents optimal performance in its evaluation metric.

Model Variant	Tumor IoU	Tumor Dice	Tumor Precision	Tumor Recall	Liver Dice	Mean Dice
Attention U-Net	61.73	76.34	77.52	75.19	94.76	90.27
+SCDA	66.52	79.89	81.94	77.95	95.37	91.67
+BiFormer	66.52	81.30	81.79	**80.82**	95.45	92.17
+MSB	67.38	80.51	82.40	78.71	95.26	91.84
SBM–Attention U-Net	**69.93**	**82.30**	**84.84**	79.92	**95.64**	**92.57**

**Table 3:** Consolidated ablation study results on the CHAOS dataset (dice). Bold represents optimal performance in its evaluation metric.

Model Variant	Liver	Right Kidney	Left Kidney	Spleen	Mean Dice
Attention U-Net	93.82	95.19	95.11	91.78	95.11
+SCDA	94.00	95.23	94.57	92.57	95.20
+BiFormer	94.30	95.06	95.19	93.71	95.58
+MSB	94.00	95.23	94.57	92.57	95.20
SBM–Attention U-Net	**94.98**	**96.41**	**95.73**	**93.73**	**96.11**

**Table 4:** Ablation study for the proposed method on 3Dircadb dataset. Bold represents optimal performance in its evaluation metric.

Architecture	Year	Core Mechanism	Tumor Dice
SBM–Attention U-Net	2026	CNN+Transformer	**87.69**
TransUNet [30]	2021	CNN+Transformer	76.06
Swin-UNet [43]	2022	Swin Transformer	71.38
MS-FANet [44]	2023	Multi-Scale Feature Attention	87.50
nnU-Net (V2) [45]	2024	Self-Configuring Baseline	82.34
E^2^Net [46]	2025	Edge-Enhanced Network	83.00
G-UNETR++ [47]	2025	Gradient-Enhanced Encoder	83.21

**Table 5:** Ablation study for the proposed method on LITS dataset. Bold represents optimal performance in its evaluation metric.

Architecture	Year	Core Mechanism	Tumor Dice	Liver Dice
SBM–Attention U-Net	2026	CNN+Transformer	82.30	95.64
TransUNet [30]	2021	CNN+Transformer	82.19	94.95
Swin-UNet [43]	2022	Pure Transformer	81.73	93.64
MS-FANet [44]	2023	Multi-Scale Feature Attention	74.20	94.80
SBCNet [48]	2024	Dual-Branch CNN	81.35	94.21
T-MPEDNet [49]	2025	CNN+Transformer	81.98	95.54
SegMamba (V2) [50]	2025	State-Space Model	**82.68**	**96.62**

**Table 6:** Ablation study for the proposed method on CHAOS dataset. Bold represents optimal performance in its evaluation metric.

Architecture	Year	Core Mechanism	Liver Dice	R. Kidney Dice	L. Kidney Dice	Spleen Dice
SBM–Attention U-Net	2026	Hybrid Attention + MSB	94.98	**96.41**	**95.73**	**93.73**
Swin-UNETR [51]	2021	Swin Transformer	87.67	86.50	85.82	86.39
MISSFormer [52]	2022	Hierarchical Transformer	91.88	93.52	92.73	91.13
SegMamba (V2) [50]	2025	State-Space Model	**95.38**	96.20	95.08	92.40
MRSegmentator [53]	2024	nnU-Net variant	93.55	92.62	90.02	89.58
CabiNet [54]	2025	Multi-Dataset Learning	90.39	87.41	89.09	90.78

**Table 7:** Comparison of model training-time on three datasets.

Dataset	Attention U-Net	+SCDA	+BiFormer	+MSB	SBM–Attention U-Net
3Dircadb	17.2 min	39.63 min	38.13 min	56.42 min	1.16 h
LITS	16.75 min	29.17 min	21.7 min	47.63 min	1.05 h
CHAOS	22.23 min	45.38 min	33.14 min	47.24 min	1.2 h

**Table 8:** Comparison of model testing time on three datasets.

Dataset	Attention U-Net	+SCDA	+BiFormer	+MSB	SBM–Attention U-Net
3Dircadb	16.33 min	37.77 min	36.64 min	52.77 min	1.1 h
LITS	15.93 min	27.97 min	20.66 min	44.76 min	1 h
CHAOS	21.08 min	43.65 min	31.47 min	43.95 min	1.15 h

## Data Availability

The original data presented in the study are openly available in 3Dircadb at https://www.ircad.fr/research/data-sets/liver-segmentation-3d-ircadb-01/ (accessed on 20 April 2025), in LITS at https://competitions.codalab.org/competitions/17094 (accessed on 20 April 2025), and in CHAOS at https://chaos.grand-challenge.org/Combined_Healthy_Abdominal_Organ_Segmentation/ (accessed on 20 April 2025).

## Supplementary Materials

The following supporting information can be downloaded at https://www.mdpi.com/article/10.3390/s26061851/s1, Table S1: Attention U-Net evaluation metrics results on the 3Dircadb dataset; Table S2: Attention U-Net evaluation metrics results on the LITS dataset; Table S3: Attention U-Net evaluation metrics results on the CHAOS dataset; Table S4: Performance of Attention U-Net with SCDA on 3Dircadb Dataset; Table S5: Performance of Attention U-Net with BiFormer on 3Dircadb Dataset; Table S6: Performance of Attention U-Net with Mix Structure Block on 3Dircadb Dataset; Table S7: Performance of Attention U-Net with three modules on 3Dircadb Dataset; Table S8: Performance of Attention U-Net with SCDA on LITS Dataset; Table S9: Performance of Attention U-Net with BiFormer on LITS Dataset; Table S10: Performance of Attention U-Net with Mix Structure Block on LITS Dataset; Table S11: Performance of Attention U-Net with three modules on LITS Dataset; Table S12: Performance of Attention U-Net with SCDA on CHAOS Dataset; Table S13: Performance of Attention U-Net with BiFormer on CHAOS Dataset; Table S14: Performance of Attention U-Net with Mix Structure Block on CHAOS Dataset; Table S15: Performance of Attention U-Net with three modules on CHAOS Dataset.
