# Supplementary material for: SBM–Attention U-Net: A Hybrid Transformer Network for Liver Tumor Segmentation in Medical Images
Source: Sensors (Basel). 2026 Mar 15;26(6):1851. doi: 10.3390/s26061851 (PMC13030151; doi:10.3390/s26061851)
Supplement: Supplementary file 1 [file sensors-26-01851-s001.zip › sensors-4116988-supplementary.pdf]

Table S1. Attention U-Net evaluation metrics results on the 3Dircadb dataset.

Class	IoU	Precision	Recall	Dice
Background	99.61	99.72	99.88	99.8
Tumor	71.48	89.35	78.13	83.37
Mean	85.54	94.54	89.01	91.58

Table S2. Attention U-Net evaluation metrics results on the LITS dataset.

Class	IoU	Precision	Recall	Dice
Background	99.43	99.74	99.69	99.72
Liver	90.03	94.35	95.17	94.76
Tumor	61.73	77.52	75.19	76.34
Mean	83.73	90.53	90.02	90.27

Table S3. Attention U-Net evaluation metrics results on the CHAOS dataset.

Class	IoU	Precision	Recall	Dice
Background	99.26	99.6	99.65	99.63
Liver	88.36	93.77	93.87	93.82
Right kidney	90.82	94.58	95.81	95.19
Left kidney	90.68	95.72	94.52	95.11
Spleen	84.8	95.12	88.66	91.78
Mean	90.79	95.76	94.5	95.11

Table S4. Performance of Attention U-Net with SCDA on 3Dircadb Dataset.

Class	IoU	Precision	Recall	Dice
Background	99.66	99.77	99.89	99.83
Tumor	75.41	90.30	82.05	85.98
Mean	87.54	95.04	90.97	92.91

Table S5. Performance of Attention U-Net with BiFormer on 3Dircadb Dataset.

Class	IoU	Precision	Recall	Dice
Background	99.67	99.8	99.87	99.84
Tumor	76.34	89.08	84.22	86.58
Mean	88	94.44	92.04	93.21

Table S6. Performance of Attention U-Net with Mix Structure Block on 3Dircadb Dataset.

Class	IoU	Precision	Recall	Dice
Background	99.68	99.8	99.88	99.84
Tumor	76.88	89.82	84.23	86.93
Mean	88.28	94.81	92.05	93.39

Table S7. Performance of Attention U-Net with three modules on 3Dircadb Dataset.

Class	IoU	Precision	Recall	Dice
Background	99.7	99.81	99.89	99.85

Tumor	78.07	90.4	85.13	87.69
Mean	88.89	95.11	92.51	93.77

Table S8. Performance of Attention U-Net with SCDA on LITS Dataset.

Class	IoU	Precision	Recall	Dice
Background	99.5	99.76	99.74	99.75
Liver	91.16	95.09	95.66	95.37
Tumor	66.52	81.94	77.95	79.89
Mean	85.72	92.26	91.11	91.67

Table S9. Performance of Attention U-Net with BiFormer on LITS Dataset.

Class	IoU	Precision	Recall	Dice
Background	99.51	99.77	99.74	99.75
Liver	91.3	95.22	95.69	95.45
Tumor	66.52	81.79	80.82	81.3
Mean	85.72	92.26	92.08	92.17

Table S10. Performance of Attention U-Net with Mix Structure Block on LITS Dataset.

Class	IoU	Precision	Recall	Dice
Background	99.48	99.76	99.72	99.74
Liver	90.94	94.81	95.7	95.26
Tumor	67.38	82.4	78.71	80.51
Mean	85.93	92.33	91.38	91.84

Table S11. Performance of Attention U-Net with three modules on LITS Dataset.

Class	IoU	Precision	Recall	Dice
Background	99.53	99.76	99.76	99.76
Liver	91.65	95.52	95.77	95.64
Tumor	69.93	84.84	79.92	82.3
Mean	87.04	93.37	91.82	92.57

Table S12. Performance of Attention U-Net with SCDA on CHAOS Dataset.

Class	IoU	Precision	Recall	Dice
Background	99.29	99.61	99.69	99.65
Liver	88.67	94.41	93.58	94
Right kidney	90.89	95.32	95.13	95.23
Left kidney	89.70	95.34	93.81	94.57
Spleen	86.17	94.63	90.6	92.57
Mean	90.94	95.86	94.56	95.2

Table S13. Performance of Attention U-Net with BiFormer on CHAOS Dataset.

Class	IoU	Precision	Recall	Dice
Background	99.33	99.67	99.65	99.66

Liver	89.21	94.03	94.57	94.3
Right kidney	90.58	94.86	95.26	95.06
Left kidney	90.82	95.89	94.5	95.19
Spleen	88.16	93.76	93.65	93.71
Mean	91.62	95.64	95.53	95.58

Table S14. Performance of Attention U-Net with Mix Structure Block on CHAOS Dataset.

Class	IoU	Precision	Recall	Dice
Background	99.29	99.61	99.69	99.65
Liver	88.67	94.41	93.58	94
Right kidney	90.89	95.32	95.13	95.23
Left kidney	89.7	95.34	93.81	94.57
Spleen	86.17	94.63	90.6	92.57
Mean	90.94	95.86	94.56	95.2

Table S15. Performance of Attention U-Net with three modules on CHAOS Dataset.

Class	IoU	Precision	Recall	Dice
Background	99.4	99.7	99.7	99.7
Liver	90.45	94.67	95.3	94.98
Right kidney	93.07	96.76	96.06	96.41
Left kidney	91.81	96.88	94.61	95.73
Spleen	88.19	94.2	93.26	93.73
Mean	92.59	96.44	95.79	96.11
